# Protective Effects of Deer Antler Peptides on D-Galactose-Induced Brain Injury

**DOI:** 10.3390/nu17142306

**Published:** 2025-07-13

**Authors:** Sihan Chen, Ying Zong, Jianming Li, Zhongmei He, Rui Du

**Affiliations:** 1 College of Chinese Medicinal Materials, Jilin Agricultural University, Changchun 130118, China; 13708045625@163.com (S.C.); zongying7699@126.com (Y.Z.); m15568781138@163.com (J.L.); 2 College of Pharmacy, Yanbian University, Yanji 133002, China

**Keywords:** aging, brain injury, D-galactose, deer antler peptides

## Abstract

**Background/Objectives:** The aim of this study was to investigate the role and potential mechanism of deer antler peptides (DAP) in D-galactose (D-gal)-induced brain injury. **Methods:** In the in vivo study, C57BL/6J mice were intraperitoneally injected with 400 mg/kg D-gal and gavaged with DAP (50 and 200 mg/kg) for 5 weeks. In vitro studies, D-gal (30 μg/mL) induced senescent BV2 cells were used for further research. **Results:** DAP increased the expression of BDNF and VEGF in the brain tissue of aging mice, reduced the levels of oxidative stress and inflammatory factors in serum, and decreased the pathological damage of brain tissue. In vitro, DAP promoted the proliferation of D-gal-induced senescent BV2 cells, reduced ROS level, and inhibited the release of IL-1β, IL-6 and TNF-α. In addition, DAP significantly reduced the protein expressions of TLR4 and MyD88, and inhibited the phosphorylation of NF-κB. **Conclusions:** DAP can inhibit the TLR4/MyD88/NF-κB signaling pathway, reduce oxidative stress and inflammation, and promote neovascularization. This indicates the therapeutic potential of DAP as a natural bioactive substance in preventing aging-related brain injury.

## 1. Introduction

Aging is a multi-faceted and inevitable natural phenomenon, refers to the structure and function of the degenerative changes. During the aging process, the nervous system gradually declines, leading to phenomena such as a decrease in learning and memory abilities [1,2], and it has the characteristic of being chronic and irreversible. As an important organ of the human body, the brain is also greatly affected by aging. The most notable aging change is the brain dysfunction marked by neurodegenerative diseases, such as Alzheimer’s disease (AD), Parkinson’s disease (PD), etc. [3]. Among them, brain injury is a common complication in the aging process, which seriously affects the daily life of patients and brings a huge burden to families [4,5,6,7,8]. Therefore, it is essential to prevent and treat brain injury caused by aging.

Deer antler peptides (DAP) is a protein polypeptide active factor obtained from the antlers of sika deer. It is a kind of antioxidant, anti-inflammatory and anti-aging properties of natural active substance [9,10,11]. At present, studies have explored the anti-aging effects of DAP. For instance, DAP can enhance the learning and cognitive functions of aging mice and exert potential neuroprotective effects in models of PD and AD [12,13,14]. Furthermore, it is reported that DAP can also improve brain injury. A study showed that DAP can reduce cerebral ischemic, mainly by inhibiting NF-κB pathway to less oxidative stress, inflammation and apoptosis [15]. Pei et al. [16] found that deer antler polypeptide PNP1 can inhibit hypoxic brain injury in MCAO model mice, mainly related to reducing tissue inflammatory factors, promoting tissue angiogenesis, and inhibiting microglial inflammatory responses. Wu et al. [17] found that PAP can treat hypoxic-ischemic encephalopathy (HIE), and this effect is related to enhancing the expression of neurotrophic factors. At present, there are relatively few existing literatures on DAP on aging-related brain injury. Therefore, it is of vital importance for us to study the role of DAP in brain injury caused by aging. This can not only reduce the incidence of brain-related diseases, but also provide new directions and ideas for the problem of brain injury related to aging.

Many studies have shown that D-galactose is a classic drug used to construct natural aging model, and this model has been widely applied in the research on age-related degeneration and organ injury [18]. Therefore, the objective of our research was to explore the neuroprotective effect of DAP on D-gal-induced brain injury in vitro and in vivo. Furthermore, we analyzed the changes in the levels of TLR4/MyD88/NF-κB pathway-related proteins under DAP intervention to clarify the potential mechanism of DAP in alleviating aging-related brain injury.

## 2. Materials and Methods

### 2.1. Materials

D-gal was purchased from Shanghai Yuanye Biology (Shanghai, China). Deer antler peptides was provided by Shaanxi Ronglin Biotechnology Co., Ltd. (Shaanxi, China). TLR4 signaling inhibitor Resatorvid (TAK-242) was purchased from MedChemExpress (Monmouth Junction, NJ, USA). brain-derived neurotrophic factor (BDNF), tumor necrosis factor-α (TNF-α), interleukin-1 β (IL-1β) and vascular endothelial growth factor (VEGF) ELISA kits and BCA kits were purchased from Boyan Biotechnology Co., Ltd. (Nanjing, China). The CCK8, NO, lactate dehydrogenase (LDH), ROS, cell proliferation, mitochondrial membrane potential detection kits were provided by Biyantian (Shanghai, China). The antibodies of TLR4, MyD88, NF-κB P65, P-NF-κB P65, Bax, GAPDH, Bcl-2 and Cleaved Caspase-3 were purchased from Wanlei Biology (Shenyang, China).

### 2.2. Animals

Forty C57BL/6J mice (25 ± 1 g) were provided by Changchun Yisi Technology (Changchun, China) for this study. This experiment has been approved by the animal rthics committee of Jilin agricultural university. The ethics review acceptance number is: 20211011003. All animals are under the condition of the standardized breeding, 12 h of light/dark cycles, temperature control in 22 ± 2 °C, relative humidity of 50 ± 10%. The mice were randomly divided into control, model (D-gal 400 mg/kg), DAP-L (50 mg/kg), DAP-H (200 mg/kg), and DAP-H+TAK-242 group (200 mg/kg DAP and 3 mg/kg TAK-242) (*n* = 8). All groups except the control group were intraperitoneally injected with D-galactose at 400 mg/kg every day. The DAP-L and the DAP-H group were administered DAP by gavage every day (50 and 200 mg/kg). The DAP-H+TAK-242 group was gavaged with 200 mg/kg of DAP every day and TAK-242 at a dose of 3 mg/kg by intraperitoneal injection once a week for 5 weeks. In the experiment, we observed and recorded the weight of mice.

### 2.3. MWM Test

The MWM test is based on previous research [19]. The water maze was set as a circular pool with a diameter of 120 cm, a height of 60 cm, and a water filling depth of 20 cm. We placed a circular platform with a diameter of 10 cm in quadrant II, and the water level was 1 cm higher than the platform. The experiment began in the 6th week. From the 1st to the 5th day, the positioning navigation experiment was conducted. All the mice were placed in the water pool from different quadrants for two minutes and trained three times a day for five consecutive days. On the sixth day, we removed the platform. The camera equipment recorded the escape latency of each mouse and the number of times the platform crossed within two minutes.

### 2.4. Immunofluorescence

Slightly modified based on the method of Qi and Hao [20,21] the expressions of BDNF and VEGF in brain tissue were detected by immunofluorescence (IF). Mice were euthanized under anesthesia, and brain tissues were removed, and fixed for 24 h. The tissue sections of 5 μm were deparaffinized. Sections were incubated with the primary antibodies of BDNF and VEGF, then incubated with the secondary antibody. Finally, DAPI staining was added, observed and photographed under a fluorescence microscope, and analyzed using Image J software (Java 1.8.0).

### 2.5. HE and Nissl Staining

The brain tissues were fixed with 4% paraformaldehyde for 24 h. The brain tissues were prepared into paraffin sections of 5 μm, stained with hematoxylin-eosin and toluidine blue respectively, and finally analyzed using Image J software (Java 1.8.0).

### 2.6. ELISA

After the water maze test, the eye blood of mice was obtained. The serum was collected at 12,000 rpm 15 min. The concentrations of IL-1β, IL-6, TNF-α, SOD, CAT and SOD were detected according to the kit instructions. The absorbance at 450 nm was obtained using an microplate reader (Bio-Rad, Hercules, CA, USA).

### 2.7. Cell Viability Detection

BV2 Cells were seeded into 96-well plates at a density of about 1 × 10^5^ cells per well. Cells were cultured with DAP at concentrations of 100, 200, 400, 600 and 800 μg/mL for 12 h. Then, CCK8 solution was added and incubated for 30 min. The absorbance at 450 nm was determined using an microplate reader (Bio-Rad, Hercules, CA, USA).

### 2.8. Determination of NO and LDH

After 24 h culture with 30 μg/mL D-gal, the cells were further cultured with DAP (200 and 600 μg/mL) for 12 h. The content of NO and LDH were measured according to theu manual.

### 2.9. ROS, Cell Proliferation, Mitochondrial Membrane Potential Detection

According to the instructions, the cells were incubated respectively with DCFH-DA, EdU dye and JC-1 staining solution at 37 °C for 30 min. Observations were made using an Axiophot microscope (Carl Zeiss, Jena, Germany) and its intensity was analyzed by Image J software (Java 1.8.0).

### 2.10. Western Blot

The lysate of mouse brain tissue (30 mg) was prepared, and then the protein concentration was determined by the BCA method. After the electric transfer, the PVDF membrane was sealed with 5% skimmed milk powder for 1 h. PVDF membrane and primary antibody were incubated overnight. The primary antibodies: TLR4 (1:2000), MyD88 (1:2000), NF-κB P65 (1:1500), P-NF-κB P65 (1:1500), Bcl-2 (1:2000), Bax (1:2000), Cleaved Caspase-3 (1:2000) and GAPDH (1:2000). Then they were incubated with the secondary antibody at room temperature for 1 h. The mean gray of protein bands were analyzed by ImageJ software (Java 1.8.0).

### 2.11. Statistical Analysis

Data were analyzed using Graphpad Prism 10.1.2 and presented as mean ± standard deviation. Statistical significance was analyzed using one-way analysis of variance (ANOVA). *p* < 0.05 indicated statistical significance.

## 3. Results

### 3.1. The Effect of DAP on Learning and Memory Ability in D-Gal-Induced Aging Mice

**Figure 1:**
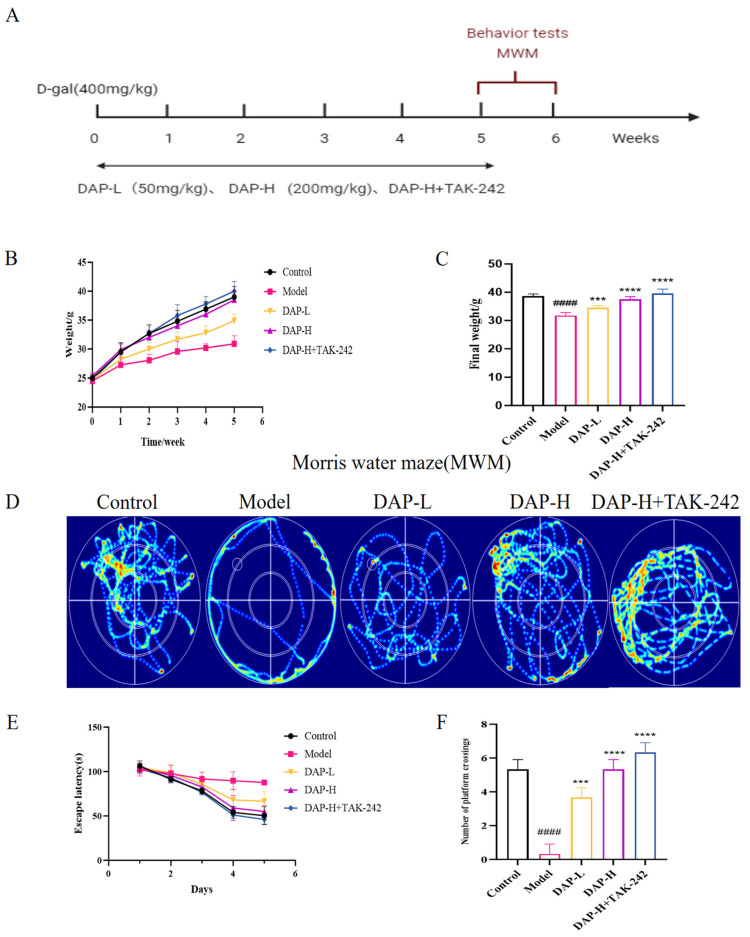
The effect of DAP on learning and memory ability in D-gal-induced aging mice. (**A**) Design process of animal experiments; (**B**) Weight changes in mice; (**C**) Final weight in mice; (**D**) The thermal infrared trajectory of MWM test; (**E**) Escape latency of all mice on days 1–5; (**F**) The number of platform crossings. *n* = 8, ^####^ *p* < 0.0001 vs. control, **** *p* < 0.0001, *** *p* < 0.001 vs. model.

During the 5-week experiment, the model group mice gained weight slowly, especially after the third week. At the end of the administration, the DAP-H and DAP-H+TAK-242 groups had significantly higher weight than the model group (Figure 1B,C). In addition, model group mice showed obvious cognitive impairment, their escape latency showed a slow downward trend with no statistical difference (Figure 1D,E), the number of times crossing the platform has decreased significantly, and the swimming distance increased. After treatment in DAP, these conditions were reversed (Figure 1F).

### 3.2. The Effect of DAP on Brain Tissue in D-Gal-Induced Aging Mice

**Figure 2:**
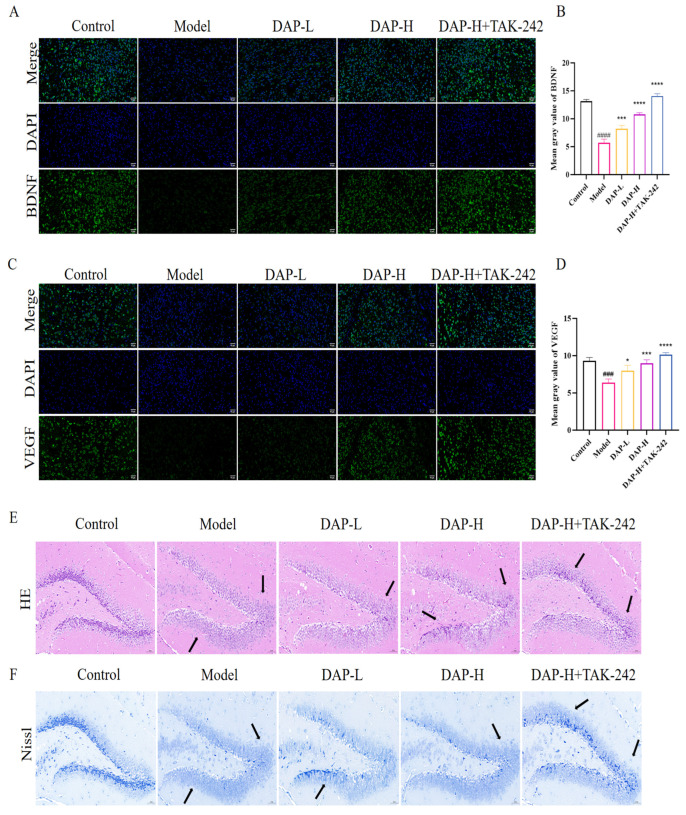
The effect of DAP on brain tissue in D-gal-induced aging mice. (**A**) Immunofluorescence results of BDNF (50 μm); (**B**) Mean gray value of BDNF; (**C**) Immunofluorescence results ofVEGF (50 μm); (**D**) Mean gray value of VEGF; (**E**) HE results (50 μm); (**F**) Nissl results (50 μm). *n* = 8, ^####^ *p* < 0.0001, ^###^ *p* < 0.001 vs. control, **** *p* < 0.0001, *** *p* < 0.001, * *p* < 0.05 vs. model.

BNDF and VEGF levels model group was obviously lower than the control group. But after the DAP processing, the expression levels increased significantly in a dose-dependent manner. In addition, the combined treatment of DAP and TAK-242 could more significantly increase the levels of BDNF and VEGF (Figure 2A–D). Figure 2E,F show the histological assessment of the brain tissues of mice. The number of neurons in the DG region of the hippocampus in the model group was significantly reduced, the structure was loose and sparsely arranged, and most of the nuclei showed nuclear condensation. This phenomenon can be significantly reversed after DAP treatment.

### 3.3. The Effect of DAP on Serum Inflammatory Factors and Antioxidant Indices in D-Gal-Induced Aging Mice

**Figure 3:**
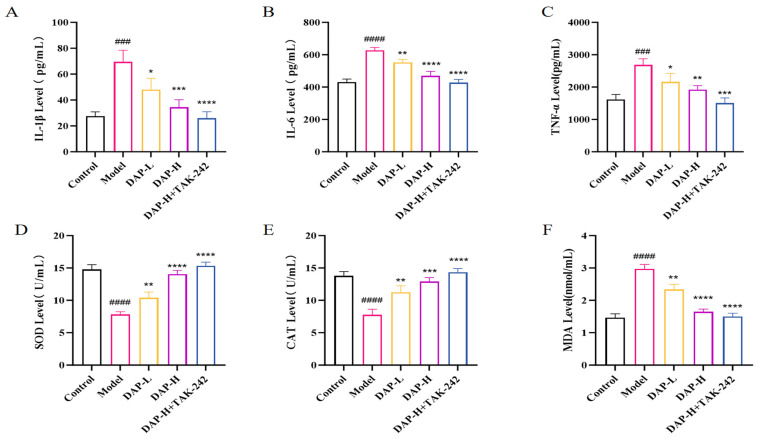
The effect of DAP on serum inflammatory factors and antioxidant indices in D-gal-induced aging mice. (**A**) IL-1β levels; (**B**) IL-6 levels; (**C**) TNF-α levels; (**D**) SOD levels; (**E**) CAT levels; (**F**) MDA levels. *n* = 8, ^####^ *p* < 0.0001, ^###^ *p* < 0.001 vs. control, **** *p* < 0.0001, *** *p* < 0.001, ** *p* < 0.01, * *p* < 0.05 vs. model.

The release of IL-1β, IL-6 and TNF-α in the model group increased significantly (Figure 3A–C). In contrast, their levels were significantly decreased in the DAP-L, DAP-H, AVP-H and TAK-242 groups, and DAP-H, AVP-H and TAK-242 showed more obvious effects. Meanwhile, the contents of SOD and CAT in the model group mice decreased significantly, and the contents of MDA increased significantly (Figure 3D–F).

### 3.4. The Effect of DAP on TLR4/MyD88/NF-κB Pathway in D-Gal-Induced Aging Mice Brain

**Figure 4:**
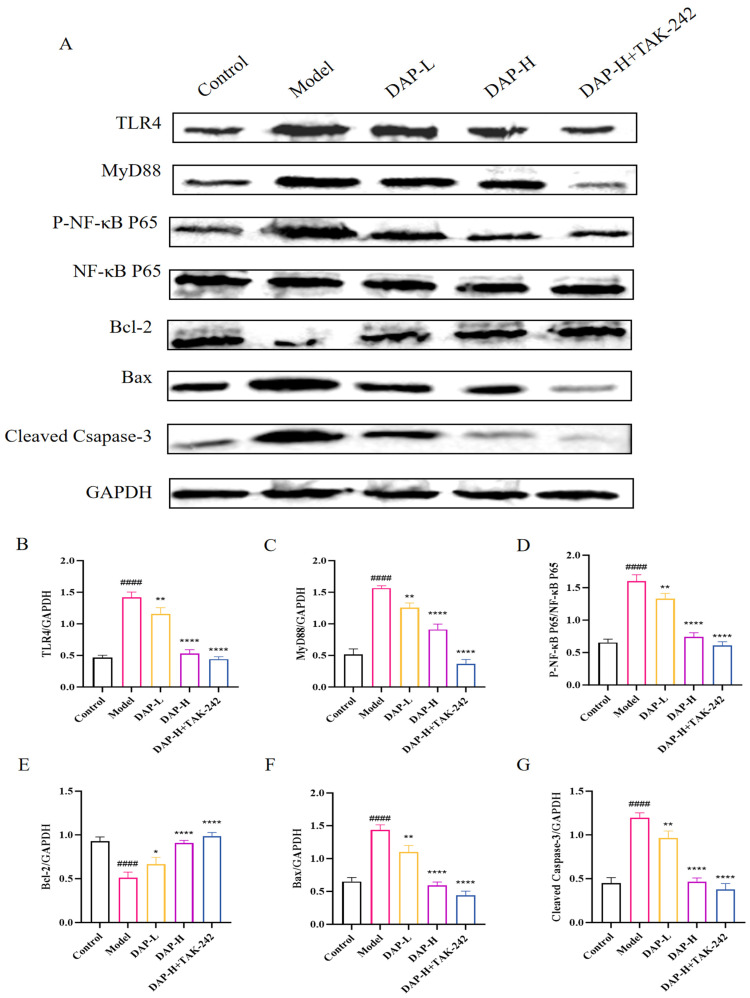
The effect of DAP on TLR4/MyD88/NF-κB pathway in D-gal-induced aging mice brain. (**A**) Western blot results; (**B**–**G**) Relative levels of TLR4, MyD88, P-NF-κB P65, Bax, Cleaved Caspase-3 and Bcl-2. *n* = 8, ^####^ *p* < 0.0001 vs. control, **** *p* < 0.0001, ** *p* < 0.01, * *p* < 0.05 vs. model.

Results of Western blot showed that DAP can significantly reduce expressions of TLR4, MyD88 and P-NF-κB P65, while the addition of TAK-242 under DAP-H treatment could further reduce their expressions. Furthermore, DAP can significantly increase expressions of Bcl-2, reduce expressions of Bax and Cleaved Caspase-3 (Figure 4A–G).

### 3.5. The Effect of DAP on the Viability and Proliferation of D-Gal-Induced BV2 Microglia

**Figure 5:**
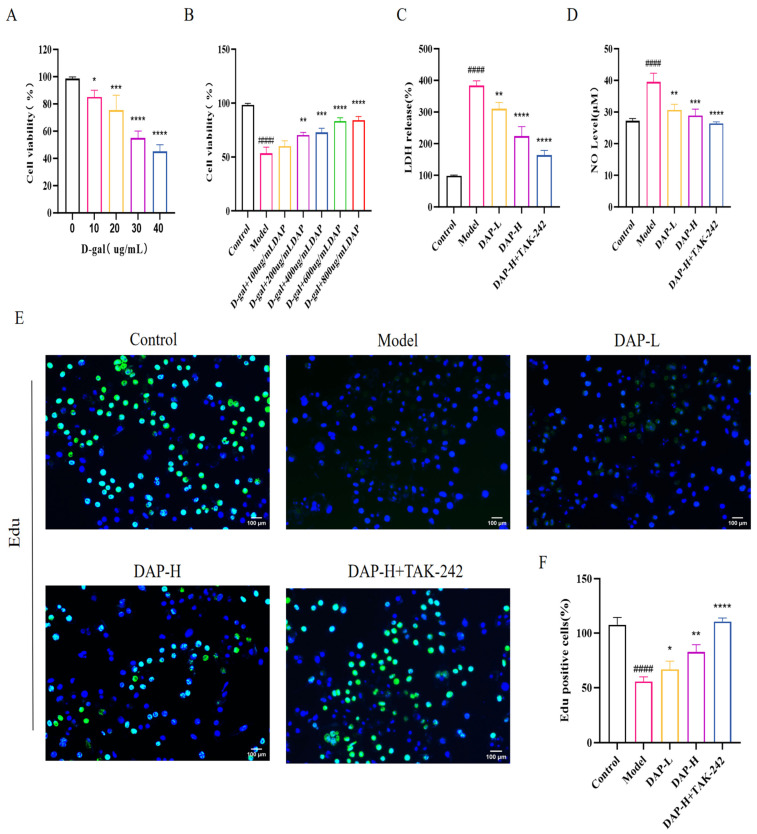
The effect of DAP on the viability and proliferation of D-gal-induced BV2 microglia. (**A**) The effect of different concentrations of D-gal on BV2 cells; (**B**) The effect of different concentrations of DAP on the viability of BV2 cells; (**C**) LDH release; (**D**) NO level; (**E**,**F**) Edu staining results (100 μm). *n* = 8, ^####^ *p* < 0.0001 vs. control, **** *p* < 0.0001, *** *p* < 0.001, ** *p* < 0.01, * *p* < 0.05 vs. model.

BV2 cells were cultured with D-gal at concentrations of 0, 10, 20, 30 and 40 μg/mL for 24 h. When the concentration of D-gal was 30 μg/mL, the cell viability decreased to 53.49%, and the cell viability was 43.25% when the concentration was 40 μg/mL. Therefore, we chose D-gal at 30 μg/mL for the subsequent experiments (Figure 5A). Afterwards, DAP of different concentrations (100, 200, 400, 600 and 800 μg/mL) was added and the culture continued for 12 h. With the increase of DAP concentration, the cell viability gradually increased, but when the concentration increased to 800 μg/mL, the cell viability showed almost no change compared with that at 600 μg/mL. Therefore, we chose 200 μg/mL as the low dose and 600 μg/mL as the high dose (Figure 5B). The contents of LDH and NO are shown in Figure 5C,D. The contents of LDH and NO in BV2 cells treated with 30 μg/mL D-gal increased significantly, while they decreased significantly after the addition of DAP treatment and even returned to the normal level. In addition, D-gal also inhibited the proliferation of BV2 cells. After treatment with DAP, this inhibition could be relieved. When 1 μmol/L TAK-242 was added under 600 μg/mLDAP treatment, the cell proliferation almost returned to the normal level at this time (Figure 5E).

### 3.6. The Effect of DAP on Pro-Inflammatory Factors of BV2 Microglia Induced by D-Gal

**Figure 6:**
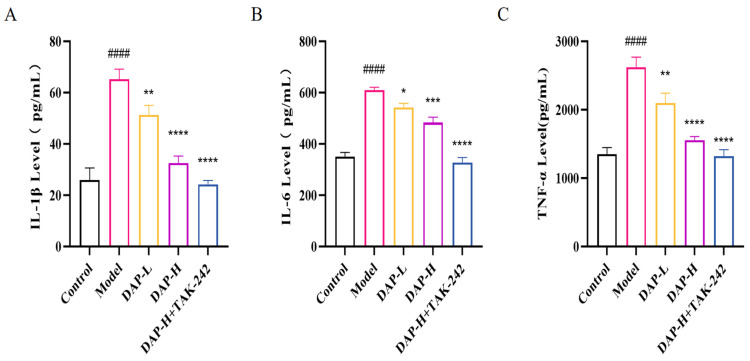
The effect of DAP on pro-inflammatory factors of BV2 microglia induced by D-gal. (**A**) IL-1β Levels; (**B**) IL-6 Levels; (**C**) TNF-α Levels. *n* = 8, ^####^ *p* < 0.0001 vs. control, **** *p* < 0.0001, *** *p* < 0.001, ** *p* < 0.01, * *p* < 0.05 vs. model.

Compared with the control group, model group of pro-inflammatory factors levels increased significantly. Their releases all decreased significantly after DAP treatment. In addition, when treated with 600 μg/mLDAP and then treated with 1 μmol/L TAK-24, the expressions of IL-1β, IL-6 and TNF-α were further decreased (Figure 6A–C).

### 3.7. The Effect of DAP on Oxidative Stress in BV2 Microglia Induced by D-Gal

**Figure 7:**
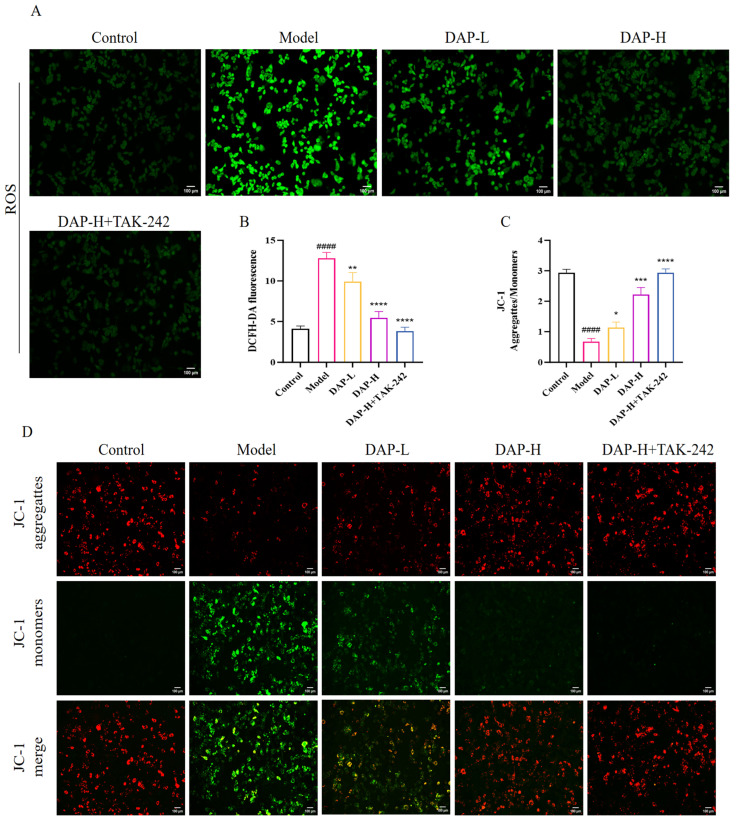
The effect of DAP on oxidative stress in BV2 microglia induced by D-gal. (**A**) ROS staining results (100 μm); (**B**) DCFH-DA fluorescence; (**C**) JC-1 Aggregattes/Monomers; (**D**) JC-1 staining results (100 μm). *n* = 8, ^####^ *p* < 0.0001 vs. control, **** *p* < 0.0001, *** *p* < 0.001, ** *p* < 0.01, * *p* < 0.05 vs. model.

Compared to the control group, ROS level in the model group increased significantly. Then, the ROS level decreased significantly after DAP treatment (Figure 7A,B). Furthermore, when DAP and TAK-242 were co-processed, ROS returned to the normal level. Meanwhile, the mitochondrial membrane potential of BV2 cells in the model group decreased significantly. When DAP was added, the mitochondrial membrane potential increased significantly. (Figure 7C,D).

### 3.8. The Effect of DAP on TLR4/MyD88/NF-κB Signaling in D-Gal-Induced BV2 Cells

**Figure 8:**
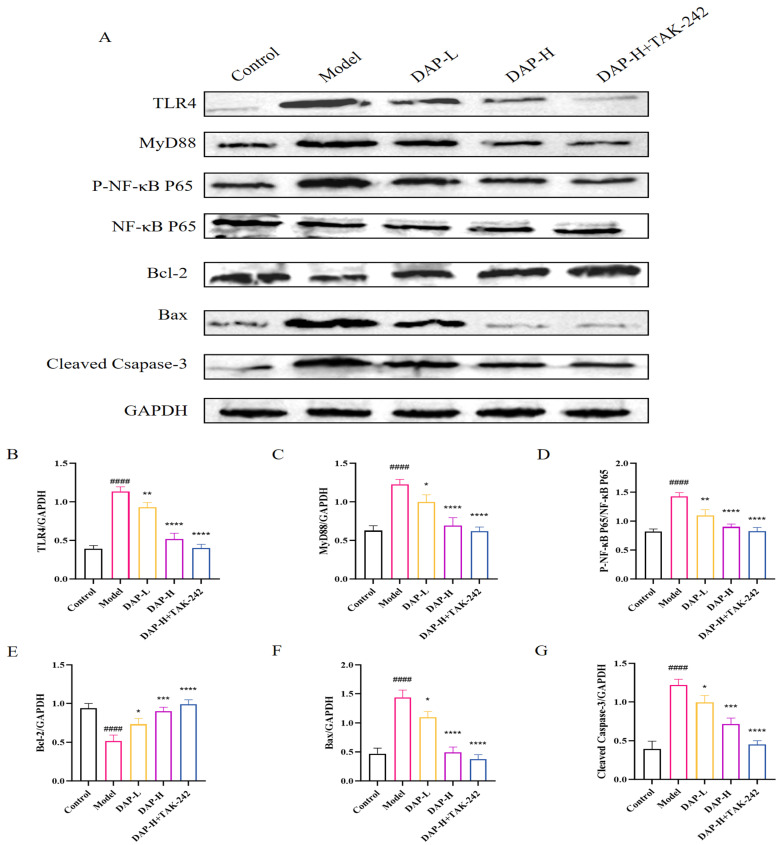
The effect of DAP on TLR4/MyD88/NF-κB signaling in D-gal-induced BV2 cells. (**A**) Western blot results; (**B**–**G**) Relative expressions of TLR4, MyD88, P-NF-κB P65, Bcl-2, Cleaved Caspase-3 and Bax,. *n* = 8, ^####^ *p* < 0.0001 vs. control, **** *p* < 0.0001, *** *p* < 0.001, ** *p* < 0.01, * *p* < 0.05 vs. model.

The expressions of TLR4, MyD88, P-NF-κB P65, Bax and Cleaved Caspase-3 in the model group significantly increased, while the protein expression of Bcl-2 significantly decreased. However, after DAP treatment, the expressions of TLR4, MyD88 and P-NF-κB P65 can be significantly reduced. Furthermore, the addition of TAK-242 under DAP-H treatment can further reduce their expression (Figure 8).

## 4. Discussion

Aging is closely related to brain-related diseases. As people age, the functions of the human brain gradually show a declining trend, which in turn leads to a significant increase in the risk of brain diseases. According to statistics, the rates of brain-related chronic diseases such as stroke, cerebral ischemia, AD and PD have gradually increased, and there is a trend of younger onset age [22,23,24]. Therefore, it is extremely urgent to explore strategies for preventing and protecting brain injury related to aging. The focus of our reseach was to explore the preventive and protective effect of DAP on aging-related brain injury using a D-gal-induced model to simulate age-related brain injury.

This study adopted the Morris water maze experiment evaluation of cognitive function in mice [25,26]. Our research found that DAP could reduce the escape latency of mice and increase the number of crossing platforms, indicating that DAP can improve memory impairment casued by D-gal in mice. BDNF is a kind of neurotrophic factor widely distributed in the brain, participating in the growth, development, survival and differentiation of neurons, and has the function of protecting nerve cells [27]. Studies have found that brain injury can induce changes in the expression of BDNF. In the early stage after injury, the expression of BDNF may temporarily decrease and then gradually increase to promote the survival and regeneration of neurons [28]. In addition, VEGF is an important factor that promotes new angiogenesis. Upregulation of VEGF expression can provide necessary nutrients and oxygen for damaged neurons and promote the recovery of neural function [29,30,31]. Our study found that DAP could effectively increase the levels of BDNF and VEGF, which is the same as the results of previous studies. Furthermore, we analyzed the histopathological changes of brain tissue using HE and Nissl staining. The results showed that D-gal induction caused significant damage to brain tissue, and DAP administration significantly alleviated these pathological changes, indicating its beneficial repair effect on D-Gal-induced brain injury. These results indicated that DAP has a potential role in protecting against brain injury related to aging.

Chronic inflammation and oxidative stress are the key factors leading to aging [32,33]. In the case of brain injury related to aging, the continuous release of inflammatory factors and the excessive free radicals produced by oxidative stress can seriously destroy neurons and speed up the aging process of the brain. Our study found that after DAP treatment, the expressions of Il-1β, IL-6, TNF-α and MDA were significantly reduced, SOD and CAT levels were significantly increased. In vitro experiments, we found that after DAP treatment, the contents of Il-1β, IL-6 and TNF-α in senescent BV2 cells were significantly reduced, the level of ROS was down-regulated, and the mitochondrial membrane potential increased significantly. At present, our study shows that DAP can alleviate D-gal-induced neuroinflammation and oxidative stress. Our research results, like previous studies, all indicate that DAP has significant anti-inflammatory and antioxidant activities in vivo and in vitro [10,34,35]. In conclusion, our results highlighted the potential of DAP in reducing age-related inflammation and oxidative stress as well as protecting against brain injury.

TLR4/MyD88/NF-κB pathway is a classic inflammation-related pathway and plays an important role in the mediation of inflammation [36]. The activation of the TLR4 signaling pathway triggers an inflammatory response that generates many inflammatory cytokines, ROS, and other substances [37,38]. These inflammatory mediators can affect the expressions and activities of Bcl-2 and Bax, thereby influencing cell apoptosis [39,40]. Our research found that compared with the model group, the expressions of TLR4, MyD88, P-NF-κB P65, Bax and Cleaved Caspase-3 were decreased after DAP treatment. After high-dose DAP treatment and administration of the TLR4 receptor inhibitor TAK-242, the expressions further decreased. The results indicated that DAP might inhibit neuroinflammation and apoptosis by regulating the TLR4/MyD88/NF-κB pathway.

Although studies have shown that DAP can prevent brain damage caused by D-gal, some limitations need to be noted. Firstly, we only used D-gal to induce specific animal and cellular aging models, which is not applicable to all aging models. Moreover, the number of experimental animals and the experimental period are limited, and it cannot simulate natural human aging. In addition, this study mainly focuses on the short-term gavage therapeutic effect of DAP, and its long-term therapeutic effect still needs further exploration. Moreover, whether other administration routes of DAP are effective still requires further research. Future research should address these limitations to gain a deeper understanding of the brain-protective effect of DAP.

## 5. Conclusions

To sum up, our study found that DAP can inhibit the TLR4/MyD88/NF-κB pathway to lower the levels of inflammatory factors and inhibit oxidative stress, thus improving the brain injury induced by D-gal. The results indicate that DAP has good potential in the prevention and treatment of brain injury caused by aging.

## Data Availability

The data provided in this study are included in this article. If you need further information, please contact the corresponding authors.
